# Mesenchymal stem cell-derived exosome alleviates sepsis- associated acute liver injury by suppressing MALAT1 through microRNA-26a-5p: an innovative immunopharmacological intervention and therapeutic approach for sepsis

**DOI:** 10.3389/fimmu.2023.1157793

**Published:** 2023-06-15

**Authors:** Jizhen Cai, Da Tang, Xiao Hao, Enyi Liu, Wenbo Li, Jian Shi

**Affiliations:** ^1^ Department of Critical Care Medicine and Hematology, The Third Xiangya Hospital, Central South University, Changsha, China; ^2^ Department of General Surgery, The Third Xiangya Hospital, Central South University, Changsha, China; ^3^ Department of Hematology, Xiangya Hospital, Central South University, Changsha, China; ^4^ Key Laboratory of Sepsis Translational Medicine of Hunan, Central South University, Changsha, China; ^5^ Department of Plastic and Aesthetic (Burn) Surgery, The Second Xiangya Hospital of Central South University, Changsha, China

**Keywords:** sepsis, MALAT1, exosome, immunopharmacological interventions, immune regulation, mesenchymal stem cells, miR-26a-5p

## Abstract

**Background:**

Sepsis is a syndrome with the disturbed host response to severe infection and is a major health problem worldwide. As the front line of infection defense and drug metabolism, the liver is vulnerable to infection- or drug-induced injury. Acute liver injury (ALI) is thus common in patients with sepsis and is significantly associated with poor prognosis. However, there are still few targeted drugs for the treatment of this syndrome in clinics. Recent studies have reported that mesenchymal stem cells (MSCs) show potential for the treatment of various diseases, while the molecular mechanisms remain incompletely characterized.

**Aims and Methods:**

Herein, we used cecal ligation puncture (CLP) and lipopolysaccharide (LPS) plus D-galactosamine (D-gal) as sepsis-induced ALI models to investigate the roles and mechanisms of mesenchymal stem cells (MSCs) in the treatment of ALI in sepsis.

**Results:**

We found that either MSCs or MSC-derived exosome significantly attenuated ALI and consequent death in sepsis. miR‐26a‐5p, a microRNA downregulated in septic mice, was replenished by MSC-derived exosome. Replenishment of miR‐26a‐5p protected against hepatocyte death and liver injury caused by sepsis through targeting Metastasis Associated Lung Adenocarcinoma Transcript 1 (MALAT1), a long non-coding RNA highly presented in hepatocyte and liver under sepsis and inhibiting anti-oxidant system.

**Conclusion:**

Taken together, the results of the current study revealed the beneficial effects of MSC, exosome or miR-26a-5p on ALI, and determined the potential mechanisms of ALI induced by sepsis. MALAT1 would be a novel target for drug development in the treatment of this syndrome.

## 1 Introduction

Sepsis is a life-threatening syndrome secondary to severe infection, accounting for a majority of death in critical-care patients (1, 2). With the development of multi-drug resistant bacteria and the boost of the aging population, the incidence of sepsis has been increasing in recent decades (3). Although the advance in antibiotics and life-supporting techniques allows a decreasing mortality rate, therapeutic strategies are still limited in clinics for the treatment of sepsis.

As the frontier of infection defense, the liver has a central role during sepsis and is essential for the regulation of immune defense and drug metabolism (4, 5). The liver could be activated by microbes or alarm signals, and thus releases pro-inflammatory cytokines and recruits immune cells, facilitating the clearance of microbes. In addition, the liver, with the interaction of parenchymal and non-parenchymal cells, modulates the inflammatory response to infection so as to maintain immunological homeostasis (4, 5). However, the liver is also a target for sepsis-related injury, where excessive microbes and cytokine storm induced by severe infection can over-activate inflammatory response and interrupt the homeostasis, thereby rendering hepatocytes to be hyperactive or fall into irreversible death (6, 7). Acute liver injury (ALI) has been reported to be a common complication of sepsis, occurring in approximately 50% of septic patients (8). Sepsis is the most common trigger for liver failure and results in increased mortality. Acute liver dysfunction substantially impairs the prognosis of sepsis and serves as a powerful independent predictor of mortality in the intensive care unit. Therefore, it is urging to explore novel methods or drugs for rescuing ALI in the treatment of sepsis.

Mesenchymal stem cell (MSC) is a type of pluripotent cell, characterized by excellent self-renewing and immunomodulatory properties. MSCs are widely applied in a series of preclinical studies for the treatment of tissue injury and inflammatory diseases, such as central nerve injury, graft-versus-host disease, lupus, and so on (8). MSCs have also been reported to show potential for the treatment of sepsis (9). However, potential tumorigenic properties, quality variation and undesirable side effects in hyperinflammatory circumstance limit the application of MSCs (10, 11). Alternatively, MSCs-derived exosome, a cell-free particle carrying majority of the modulatory function of MSCs, shows high potential efficacy in preclinical studies. It has been demonstrated that MSC-derived exosome protects against ischemia-reperfusion-induced liver injury by inhibiting the release of inflammatory cytokines or alarming stimuli, such as TNFα, IL-6 and high mobility group box 1 (HMGB1) (12). Above all, MSCs or MSC-derived exosome play vital roles and present promising diagnostic and therapeutic targets in organ injury.

Exosome exerts its function by transferring and releasing the contents, including proteins, microRNA, and so on (13). MicroRNAs (miRNAs), as small noncoding RNA molecules, regulate many biological processes, such as cell proliferation, differentiation, and apoptosis by being complementary to target mRNAs, resulting in gene silence (14–16). Literature suggests that the abnormal expression level of miRNAs is relevant to the pathogenesis of various diseases (17, 18). Plasma exosomal microRNAs (miRNAs) are considered as valid circulating biomarkers for disease diagnosis and prognosis. However, the function of miRNAs from MSC-derived exosome in ALI induced by sepsis remains unclear.

Metastasis Associated Lung Adenocarcinoma Transcript 1 (MALAT1) is an evolutionally conserved long non-coding RNA, implicated in various cancer and inflammatory diseases (19). Once stimulation or subjected to environmental stress, such as oxidized-LDL, lipopolysaccharides, hypoxia and high glucose, MALAT1 is commonly upregulated in different cells, thus inducing pro-inflammatory cytokine release, inflammatory cell infiltration and cell death, thereby facilitating inflammatory responses and tissue injury (20–22). Herein, we discovered that MSCs, MSCs-derived exosome, and miR-26a-5p have the capacity to protect against sepsis-associated ALI. Using genetically modified mice, we identified that this protection could be mainly attributed to the inhibition of MALAT1 *via* the exosome-delivered miR-26a-5p.

Thus, the findings of the present study suggest that MSCs, MSC-derived exosome and miR-26a-5p are potential therapeutics, and MALAT1 is a promising target for the treatment of ALI in sepsis, which provides a new avenue and new insights for rescuing the syndrome.

## 2 Materials and methods

### 2.1 Mice

*Malat1* ^-/-^ and WT mice on a C57BL/6J background were purchased from Gempharmatech Co., Ltd and Hunan SJA Laboratory Animal Company, respectively. Animals were maintained in a specific pathogen-free environment at the Department of Laboratory Animals of Central South University. Male mice with an age of 8 weeks and a body weight of 22-25g were used in the present study. Free access to water and standard chow was provided to all mice during the whole experiment process. The experiment was conducted in a standard condition with a room temperature of 22-25°C and 12/12h light-dark cycles after obtaining the approval of the research ethics committee of Central South University.

### 2.2 Cecum ligation and puncture

We followed the previously reported method to conduct CLP model for polymicrobial sepsis. In brief, mice were anesthetized and the lower abdomen was split for exposing the cecum which was subsequently ligated (75%) and punctured using an 18-gauge needle. Afterward, the cecum was reloaded and the wound was closed, followed by a resuscitation *via* injecting warm saline subcutaneously. The plasma and the liver were then harvested 12h after the operation for further experiments. Time of death was recorded within 7 days.

### 2.3 Hepatocyte purification and culture

A collagenase perfusion technique was used for the purification of primary mouse hepatocytes as shown previously (19). In brief, pre-wormed PBS was injected *via* the inferior vena cava, followed by perfusion using collagenase type IV. The liver was then dissociated for releasing hepatocytes. After removing debris by a passthrough *via* a 70 μm strainer (Falcon), hepatocytes were purified by a following centrifuge (50g) using 40% percoll (Sigma-Aldrich), and plated on collagen-coated plates in Williams E medium (Sigma) containing 5% fetal bovine serum (FBS). The primary mouse hepatocytes were treated with LPS (1ug/ml) plus D-gal (10mM) for 24h. Cell viability was assessed using CCK8 (Ck04, Dojindo). In some experiments, cell lysates and supernatants were collected at indicated time points for RNA, ELISA, Cytochrome P450 activity and albumin release assay.

### 2.4 Liver dysfunction and oxidative stress

Liver dysfunction was monitored by the augment of plasma levels of aspartate aminotransferase (AST) and alanine aminotransferase (ALT), which were determined by an automatic biochemical analyzer (Sysmex DRICHEM3500, Fuji, Japan) according to the manufacturer’s instructions. For determining oxidative stress, liver homogenate in PBS was prepared and the levels of GSH and MDA were assessed using commercial kits (A006-2-1 and A003-1-2, Nanjing Jiancheng Bioengineering Institute). Albumin and Cytochrome P450 concentration in the supernatant of primary hepatocytes were analyzed using ELISA kits according to the manufacturer’s instructions (AF2818-A and AF2766-A, Hunan Aifang Biological).

### 2.5 Real-time PCR

After extracted and purified by using Trizol (Life Technologies, Gaithersburg, MD), total RNA was reversely transcribed into cDNA by using Reverse Transcription Kit (Thermo Fisher Scientific). The expression levels of target genes were assessed by quantitative real-time polymerase chain reaction (qRT-PCR) using qPCR mix (Vazymebiotech, China) and designed primers, in which β-actin was used as the endogenous control. For determining the level of microRNA, qRT-PCR miRNA Detection kit (RiboBio, China) was applied and U6 snRNA was used as the endogenous control.

### 2.6 MSCs culture and exosome purification

Human umbilical cord derived mesenchymal stem cells were cultured in Dulbecco’s modified Eagles medium containing 10% exosome-free fetal bovine serum and harvested when 90% confluence was achieved. The cells were washed and resuspended using PBS for intervention, whilst the supernatant medium was collected for exosome purification. After removing dead cells, cell debris, and large vesicles by sequential centrifugation at 200 ×g for 10 min, 2000 ×g for 20 min, and 15,000 ×g for 30 min respectively, the supernatants were centrifuged at 100,000 ×g for 80 min. Exosome was harvested by collecting the pellets after one time wash using PBS. The morphology and size distribution of exosome were examined by transmission electron microscopy and particle size analyzer, respectively. The purified exosome was finally identified by determining the expression of markers including CD9, CD63, and TSG101 *via* Western Blotting (Anti-CD9, anti-CD63 and anti-TSG101 antibodies were ab263019, ab134045 and ab125011 from Abcam, respectively, with a dilution of 1:1000).

### 2.7 Tissue histology

Mice were sacrificed after the sham-operation or the challenge of CLP at the target time point. The liver was dissociated and fixed in 4% paraformaldehyde overnight, followed by dehydration before being embedded in paraffin. Sections of the tissues were then obtained at a thickness of 4 μm and stuck onto glass slides. After dewaxed, the sections were handled using a standard hematoxylin & eosin (H&E) staining prior to imaging by using a microscope (Eclipse80i, Nikon). The liver damage was mainly determined by augment levels of plasma AST and ALT, with the assistance of the representative image of H&E stained histological section using optical microscopy. All slides were blindly examined in more than 5 high-power fields to assess the damage in each section.

### 2.8 Luciferase reporter assay

To confirm the target of miR-26a-5p, luciferase reporter vectors containing WT (UUACUUGA) and mutant (AAUGAACU) binding sites of MALAT1 were constructed and transfected into 293T cells using Lipofectamine™ 3000 (L3000015, Invitrogen), followed by a transfection of miR-26a-5p and mimic controls (100 nM using riboFECT™ CP, C10511-05, Ribobio). The luciferase activity of cells was determined 48 h later by using Luciferase Assay system (Promega Corporation).

### 2.9 Statistical analyses

Statistical analyses were conducted by using GraphPad Prism 7.0. Student *t*-test was used for comparison between two groups, whilst One- or Two-way ANOVA with Bonferroni’s *post hoc* test was used for comparisons among more than two groups. Association between groups was assessed using Spearman correlation. The survival rates of mice were analyzed using the log-rank test (n=10 per group). Data were expressed as mean ± standard error of the mean in six biological or three technical replications (23), and P value less than 0.05 was considered as a significant difference.

## 3 Results

### 3.1 Mesenchymal stem cells attenuate acute liver injury in sepsis model

**Figure 1:**
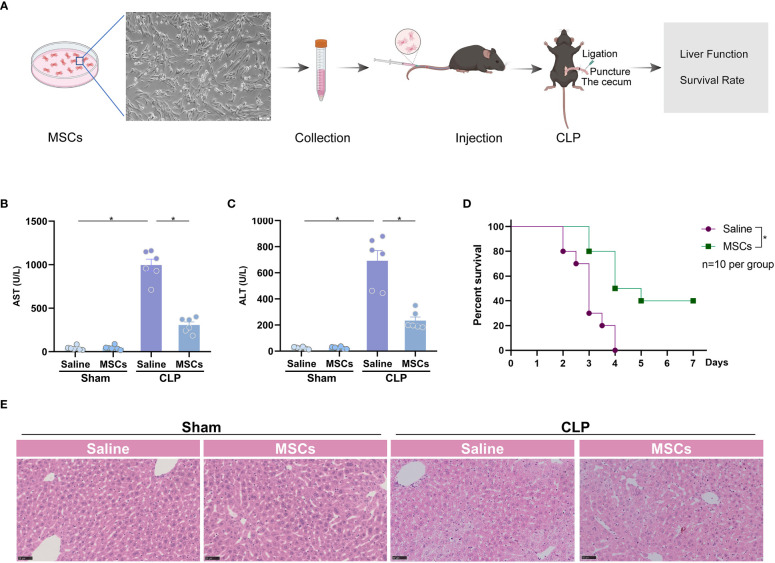
MSCs protect from sepsis-induced acute liver injury. **(A)** Schematic illustration indicating the strategy of MSCs in the treatment of CLP-induced acute liver injury. **(B, C)** Serum aspartate aminotransferase (AST) and alanine transaminase (ALT) level of mice challenged with CLP or treated with MSCs (10^6^ cells per mouse, n=6 per group). **(D)** Survival analysis of mice challenged with CLP in the presence or absence of MSCs (n=10 per group). **(E)** Representative images of H&E staining of livers from mice treated with MSCs before CLP or not (n=3 per group). Data are expressed as mean ± SEM of independent experiments analyzed by one-way ANOVA **(B, C)**, and survival curve comparison [log-rank (Mantel-Cox) test]. * Means *P*<0.05.

To investigate the role of MSCs in the treatment of sepsis-associated ALI, we administrated MSCs to mice *via* caudal vein 1h prior to the challenge of CLP, a classical model of poly-bacterial sepsis (**Figure 1A**). We found that AST and ALT, two markers of ALI, were significantly increased after challenge with CLP, indicating that sepsis induces significant liver dysfunction. The intervention of MSCs robustly reduced the augmented levels of AST and ALT (**Figures 1B, C**). Similarly, H&E staining showed that the liver was pathologically injured after CLP model, which was alleviated by the administration MSCs (**Figure 1E**). As a consequence, MSCs significantly improved the survival rate of mice in a sepsis model (**Figure 1D**). Taken together, MSCs are capable of protecting ALI in sepsis.

### 3.2 Exosome of mesenchymal stem cell exerts protective effects against sepsis-associated liver injury

**Figure 2:**
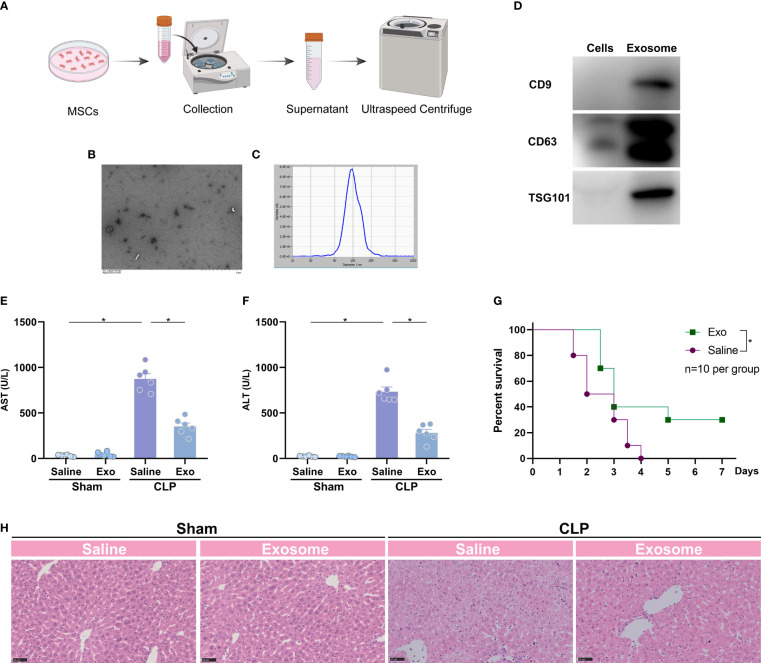
Exosome of MSCs attenuates sepsis-induced acute liver injury. **(A)** Schematic illustration indicating the purification of exosome from MSCs. **(B)** Quality of the purified exosome identified by electron microscope (white arrows). **(C)** size distribution of the purified exosome. **(D)** Expression of exosome markers, such as CD9, CD63 and TSG101, in purified exosome and MSCs. **(E, F)** Serum spartate aminotransferase (AST) and alanine transaminase (ALT) level of mice challenged with CLP or treated with the purified exosome (exosome from 5×10^6^ cells per mouse, n=6 per group). **(G)** Representative images of H&E staining of the liver from mice challenged with CLP or treated with the purified exosome (n=3 per group). **(H)** Survival analysis of mice challenged with CLP in the presence or absence of the purified exosome (n=10 per group). Data are expressed as mean ± SEM of independent experiments analyzed by one-way ANOVA **(E, F)**, and survival curve comparison [log-rank (Mantel-Cox) test]. * Means *P*<0.05.

Exosome is a functional compartment of stem cells in various physiological circumstances (13). To investigate whether protective effects of MSCs are attributed to exosome, we collected and purified exosome from the medium of cultured MSCs (**Figure 2A**), and subjected mice to CLP model 1h after the treatment of the MSCs-derived exosome (exosome from 5×10^6^ cells per mouse). Electron microscope showed regular spherical particles with a normal distributed size of around 100 nm for the purified exosome (**Figures 2B, C**). Three markers of exosome, such as CD9, CD63 and TSG101, were detectable as shown by western blotting (**Figure 2D**). The purified exosome was subsequently administrated to mice 1 h prior to a challenge of CLP. The results suggested that the exosome significantly reduced CLP-augmented AST and ALT (**Figures 2E, F**). H&E staining also pointed out a similar trend, in which the liver injury was significantly attenuated by administration of exosome (**Figure 2G**). As a result, the intervention of the exosome ameliorated CLP-induced death (**Figure 2H**). Thus, the exosome is the main executor of MSCs in the inhibition of sepsis-associated ALI.

### 3.3 Exosome of mesenchymal stem cell replenishes sepsis-downregulated miR-26a-5p in the liver

**Figure 3:**
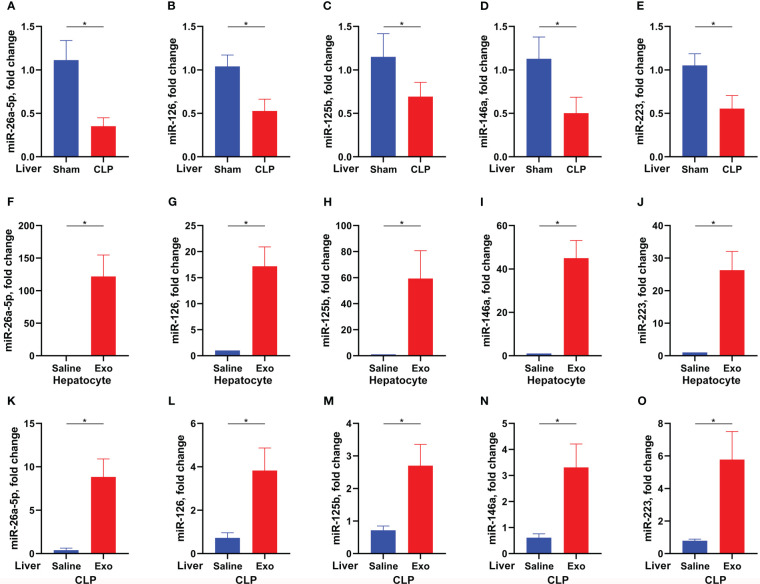
Exosome replenishes sepsis-downregulated microRNA. **(A–E)** Levels of miR-26a-5p **(A)**, miR-126 **(B)**, miR-125b **(C)**, miR-146 **(D)** and miR-223 **(E)** in the liver of mice challenged with CLP or not (n=6 per group). F-J. Levels of miR-26a-5p **(F)**, miR-126 **(G)**, miR-125b **(H)**, miR-146 **(I)** and miR-223 **(J)** in the hepatocytes treated with or without MSC-derived exosome (exosome from 1×10^5^ cells per ml, 3 technical repeats). **(K–O)** Levels of miR-26a-5p **(K)**, miR-126 **(L)**, miR-125b **(M)**, miR-146 **(N)** and miR-223 **(O)** in the liver of mice challenged with CLP and treated with or without MSC-derived exosome (exosome from 5×10^6^ cells per mouse, n=6 per group). Data are expressed as mean ± SEM of independent experiments analyzed by student *t* tests. * Means *P*<0.05.

Exosome exerts its function by transferring and releasing the contents, including proteins, microRNA, and so on (13). MicroRNA is a type of RNA around 22nt mediating target mRNA degradation and thus silencing the expression of target genes. To investigate whether MSC-derived exosome inhibits sepsis-associated ALI by microRNA and, if so, which microRNA is involved, we determined the expression of sepsis-associated microRNA in the liver of CLP-challenged mice (24). It was found that miR-26a-5p, miR-126, miR-125b, miR-146a and miR-223 were significantly downregulated in the liver after the challenge of CLP (**Figures 3A–E**). Besides, the five microRNAs were significantly upregulated in the primary hepatocytes after treated with MSC-derived exosome, with the highest expression of miR-26a-5p (**Figures 3F–J**), indicating that microRNA can be transferred into primary hepatocytes *via* MSC-derived exosome. In line with the cellular observation, we found that the five microRNA were replenished in the liver after administrated with MSC-derived exosome in the CLP model (**Figures 3K–O**). These data demonstrate that MSC-derived exosome resumes miR-26a-5p that is reduced by sepsis in the liver.

### 3.4 MiR-26a-5p alleviates sepsis-associated acute liver injury

**Figure 4:**
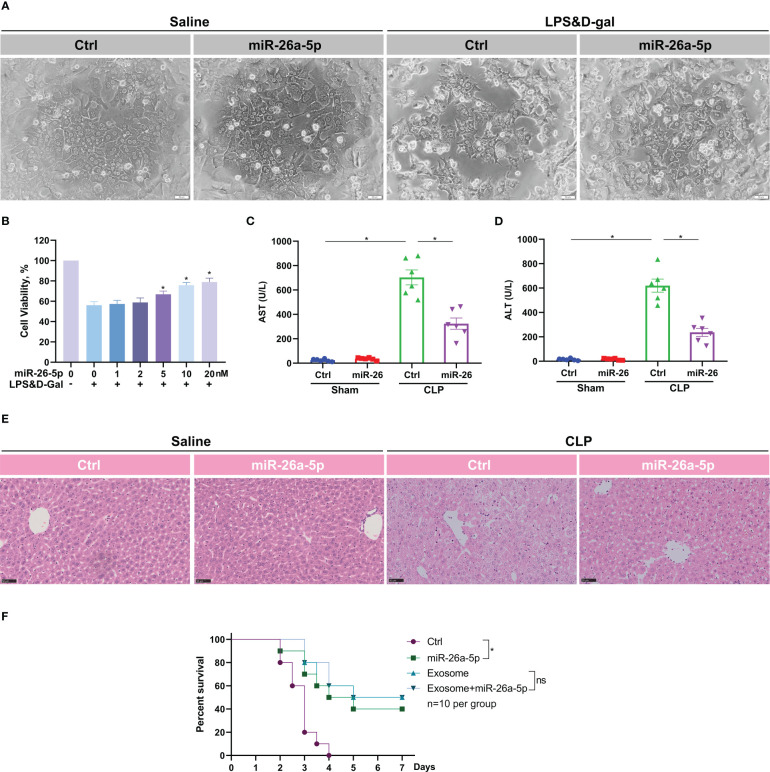
miR-26a-5p alleviates hepatocyte death and sepsis-induced acute liver injury. **(A)** Hepatocytes challenged with LPS plus D-gal or not in the presence and absence of miR-26a-5p (n=3 repeats). **(B)** Cell viability of hepatocytes challenged with LPS plus D-gal or not in the presence of various dose of miR-26a-5p (n=3 repeats). **(C, D)** Serum AST and ALT level of mice challenged with CLP or treated with the miR-26a-5p or corresponding mimic control RNA (10 nmol per mouse, n=6 per group). **(E)** Representative images of H&E staining of livers from mice treated with miR-26a-5p or corresponding mimic control RNA after CLP or not (n=3 per group). **(F)** Survival analysis of mice challenged with CLP and treated with the miR-26a-5p, corresponding mimic control RNA and/or MSC-derived exosome (n=10). Data are expressed as mean ± SEM of independent experiments analyzed by one-way ANOVA **(B, C)**, and survival curve comparison [log-rank (Mantel-Cox) test]. * Means *P*<0.05.

MiR-26a-5p is reported to function in a series of physiological processes (25). To confirm the role of miR-26a-5p in ALI, we transfected the microRNA into murine hepatocytes before a challenge of LPS plus D-gal. It was discovered that LPS/D-gal dramatically induced hepatocyte death, which was significantly reversed by the addition of miR-26a-5p (**Figure 4A**). In addition, the reversion was in a dose-dependent manner (**Figure 4B**), indicating the essential role of miR-26a-5p in the protection against hepatocyte death caused by ALI. Consistent with the cell observations, administration of miR-26a-5p attenuated CLP-induced liver injury (**Figures 4C–E**), and rescued approximately 40% of the lethality (**Figure 4F**). In line with these observation, administration of exosome alone or combining miR-26a-5p with exosome rescued approximately 50% of the lethality, which was a little higher than miR-26a-5p alone, but none of statistical significance (**Figure 4F**). Thus, these findings support that the protective effect of MSC-derived exosome against ALI is primarily mediated by miR-26a-5p in sepsis.

### 3.5 MALAT1 is the target of miR-26a-5p in acute liver injury

**Figure 5:**
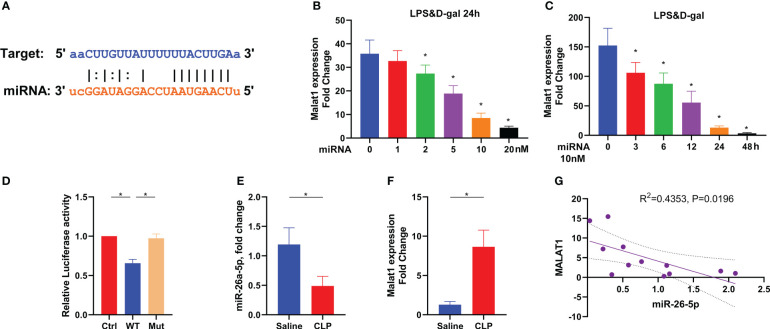
Target of miR-26a-5p in the treatment of sepsis-induced acute liver injury. **(A)** Sequence align between miR-26a-5p and MALAT1 by using ENCORI. **(B)** Levels of MALAT1 in the hepatocyte challenged with LPS&D-gal and treated with various doses of miR-26a-5p for 24 h (n=3). **(C)** Levels of MALAT1 in the hepatocyte challenged with LPS&D-gal and treated with miR-26a-5p (single dose, 10 nM) at various time points (n=3). **(D)** Luciferase activity in 293T cells transfected with miR-26a-5p and luciferase vectors containing WT and mutant target site of MALAT1 (n=3). **(E–G)** Levels of miR-26a-5p **(E)** and MALAT1 **(F)** in the liver of mice challenged with CLP or not, and the association between each other by using Spearman correlation **(G)** (n=6 per group). Data are expressed as mean ± SEM of independent experiments analyzed by one-way ANOVA **(B–F)**. * Means *P*<0.05.

To reveal the target of miR-26a-5p, we used Encyclopedia of RNA Interactomes (ENCORI) to conduct a screening. We found that MALAT1, a well-known long non-coding RNA, showed a high binding score with miR-26a-5p (**Figure 5A**). LPS plus D-gal stimulate increased MALAT1 transcription in hepatocytes, which could be restored in a dose-dependent and time-dependent manner by transfection of miR-26a-5p (**Figures 5B, C**). Mutation of the putative target site of MALAT1 prevented the reduction of luciferase activity through the specific binding site of miR-26a-5p induced by LPS plus D-gal stimulation (**Figure 5D**). In line with these observations, further *in-vivo* study revealed that miR-26a-5p was significantly dampened when MALAT1 was robustly boosted in the liver of mice challenged with CLP (**Figures 5E, F**). In addition, the downregulation of miR-26a-5p was significantly associated with the upregulation of MALAT1 (**Figure 5G**). Thus, MALAT1 is the target of miR-26a-5p in the treatment of sepsis-associated ALI.

### 3.6 MiR-26a-5p suppresses acute liver injury in sepsis by silencing MALAT1

**Figure 6:**
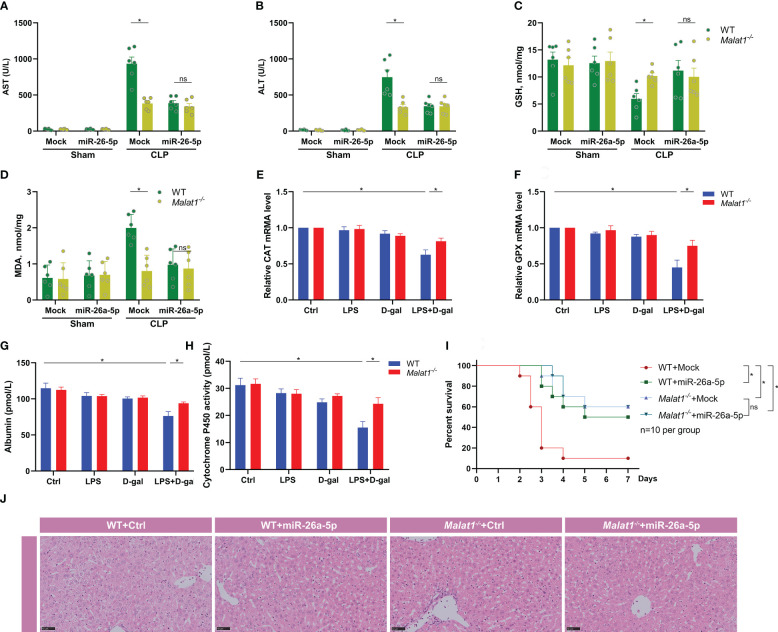
miR-26a-5p suppresses sepsis-induced acute liver injury by inhibiting MALAT-1-associated oxidative stress. **(A, B)** Serum aspartate aminotransferase (AST) and alanine transaminase (ALT) level of WT or *Malat1^-/-^* mice challenged with CLP or treated with the miR-26a-5p (n=6 per group). **(C, D)** GSH and MDA activity in the liver of WT or *Malat1^-/-^* mice challenged with CLP or treated with the miR-26a-5p (n=6 per group). **(E, F)** Quantitative real-time PCR (qRT–PCR) analysis of Catalase (Cat, **E**) and glutathione peroxidase (GPX, **F**) in primary hepatocytes (n=3 repeats). **(G, H)** Albumin **(G)** and Cytochrome P450 activity **(H)** in the supernatant of primary hepatocytes (n=3 repeats). **(I)** Survival analysis of WT or *Malat1^-/-^* mice challenged with CLP and treated with or without miR-26a-5p (n=10 per group). **(J)** Representative images of H&E staining of livers from WT or *Malat1^-/-^* mice challenged with CLP and treated with or without miR-26a-5p. Data are expressed as mean ± SEM of independent experiments analyzed by two-way ANOVA **(A–F)**, and survival curve comparison [log-rank (Mantel-Cox) test]. * Means *P*<0.05.

To identify the role of MALAT1 and miR-26a-5p in sepsis-associated ALI, we subjected WT or *Malat1*-deficient mice with CLP in the presence or absence of miR-26a-5p. We found that MALAT1 deficiency significantly reduced the CLP-augmented plasma levels of AST and ALT, indicating recovery of liver dysfunction (**Figures 6A, B**). Administration of miR-26a-5p did not additionally reduce the level of AST and ALT in *Malat1*-deficient mice (**Figures 6A, B**). A recent study reported that MALAT1 inhibits anti-oxidant system and thus promotes sepsis in CLP model (26). To investigate the mechanism of MALAT1 involving sepsis-associated ALI, we determined MDA and GSH levels in the liver of mice. The result demonstrated that CLP boosted the levels of MDA, and reduced the level of GSH in the liver, which was significantly attenuated by deleting *Malat1* without further reduction by administrating miR-26a-5p (**Figures 6C, D**). We also extracted primary hepatocytes from WT and Malat1-deficient mice to detect indicators of oxidative stress. The role of the radical scavenger enzymes catalase (Cat) and glutathione peroxidase (GPX), which remove oxygen radicals, has been assessed in this context. In our study, we provide evidence that Cat and GPX expression was diminished at the RNA level in primary hepatocytes after challenge with LPS plus D-gal, suggesting impaired detoxification of ROS, which were all attenuated by MALAT1 deficiency (**Figures 6E, F**). Albumin and Cytochrome P450 activity were analyzed to measure the link between hepatocytes function and oxidative stress. We found that the decreased albumin and impaired activity of Cytochrome P450 activity were significantly restored by deleting MALAT1 (**Figures 6G, H**), indicating that the oxidative stress contributes to the enhancement of liver damage by MALAT1. Moreover, Malat1 deficiency dramatically rescued CLP-induced mice death (**Figure 6I**) and liver injury (**Figure 6J**), and administration of miR-26a-5p did not provide further prevention on the basis of MALAT1 deficiency (**Figure 6G**). These data demonstrate that miR-26a-5p inhibits sepsis-induced ALI by targeting MALAT1, which inhibits anti-oxidant system.

## 4 Discussion

Liver injury is a common complication of sepsis, contributing to the pathogenesis of multiple organ dysfunction and predicting poor outcomes (27). However, the specific treatment targeting sepsis-associated ALI is scant in clinics. In the present study, we discovered a novel role of MSCs and MSC-derived exosome for treating ALI in sepsis, and revealed the underlying mechanism that exosome-delivered miR-26a-5p attenuate the syndrome by silencing MALAT1.

Based on the understanding of the mechanisms, a series of preclinical studies or clinical trials have investigated the effects of anti-inflammation and anti-death medicine in the treatment or prevention of liver failure in sepsis. It has been reported that pyroptosis commonly occurring in infection-induced liver injury, is a therapeutic target for the treatment of this syndrome. As important inhibitors of pyroptosis, AC-YVAD-CMK and/or Glyburide display a protective effect against liver injury in sepsis model (28).

MSCs are widely investigated in the treatment of tissue repair and various inflammatory diseases due to the excessive renewing and immunomodulatory properties (10). MSC-derived exosome suppresses pro-inflammatory cytokines and promotes anti-inflammatory cytokines and responses in a concanavalin-A-induced liver injury model (29). In addition, the particle also exerts anti-oxidant and anti-apoptotic properties, thereby attenuating liver failure and improving survival rate in mice challenged by D-galactosamine plus TNFα (30). In another liver injury model induced by carbon tetrachloride (CCL4), MSC-derived exosome delivers glutathione peroxidase 1 (GPX1) to the liver, which consequently alleviates oxidative stress and hepatic injury (31). Nevertheless, it is still unknown whether MSC-derived exosome has a protective effect on sepsis-induced liver injury. In line with previous study, we herein found that the exosome dramatically prevented hepatocyte death, liver injury and so-caused mice mortality by inhibiting MALAT1 *via* delivery of miR-26a-5p.

MALAT1 is an evolutionally conserved long non-coding RNA, implicated in various cancer and inflammatory diseases (19). It has been reported that MALAT1 is highly expressed in septic patients or mice with sepsis model (26). Depletion of MALAT1 significantly inhibited sepsis-induced mice death by improving the anti-oxidant capacity of glutathione (26). Consistently, our study found that sepsis boosts oxidative stress in the liver, thereby facilitating hepatocyte death and liver injury. MALAT1 deficiency dramatically inhibits oxidative stress, and thus attenuates liver injury in sepsis. MSC-derived exosome delivered miR-26a-5p mediated MALAT1 breakdown and consequently protected sepsis-associated ALI. Thus, silencing MALAT1 by miR-26a-5p from MSC-derived exosome ameliorates liver injury in sepsis.

In conclusion, MSCs, MSCs-derived exosome and miR-26a-5p could effectively protect against sepsis-induced ALI by inhibiting MALAT1 and MALAT1-enhanced oxidative stress. Our findings provide a new insight for understanding the molecular mechanism of ALI, and provide a potential approach and drug target for the treatment of liver injury in sepsis. However, this was a preliminary study and the pathogenesis of ALI in sepsis is complex. Therefore, large-scale studies will be needed to confirm the detailed mechanisms of MALAT1, miR-26a-5p, MSCs-derived exosome, and MSCs pathway in ALI injury of sepsis through animal experiments and clinical tests in the future.

## Data availability statement

The original contributions presented in the study are included in the article/supplementary material. Further inquiries can be directed to the corresponding author.

## Ethics statement

The animal study was reviewed and approved by Central South University.

## Author contributions

JC and DT conducted the experiments, analyzed the data and composited the figures. HX and EL assisted with experiment operation, data analysis and figure composition. EL monitored the study and wrote the first version of the manuscript. JS designed the study. JS and EL contributed to the conceptualization, data curation. JC, DT, WL and JS made the revision and the final version write-up. All authors contributed to the article and approved the submitted version.
